# Response Surface Methodology for Optimization of Hydrogel-Forming Microneedles as Rapid and Efficient Transdermal Microsampling Tools

**DOI:** 10.3390/gels9040306

**Published:** 2023-04-06

**Authors:** Jiraporn Leanpolchareanchai, Nantana Nuchtavorn

**Affiliations:** 1 Department of Pharmacy, Faculty of Pharmacy, Mahidol University, 447 Sri Ayudhaya Rd., Rajathevee, Bangkok 10400, Thailand; jiraporn.lea@mahidol.ac.th; 2 Department of Pharmaceutical Chemistry, Faculty of Pharmacy, Mahidol University, 447 Sri Ayudhaya Rd., Rajathevee, Bangkok 10400, Thailand

**Keywords:** response surface methodology, hydrogel, microneedles, interstitial fluid, transdermal, microsampling

## Abstract

Microneedles (MNs) have shown a great potential for the microsampling of dermal interstitial fluid (ISF) in a minimally invasive manner for point-of-care testing (POCT). The swelling properties of hydrogel-forming microneedles (MNs) allow for passive extraction of ISF. Surface response approaches, including Box-Behnken design (BBD), central composite design (CCD), and optimal discrete design, were employed for the optimization of hydrogel film by studying the effects of independent variables (i.e., the amount of hyaluronic acid, Gantrez^TM^ S-97, and pectin) on the swelling property. The optimal discrete model was selected to predict the appropriate variables, due to the good fit of the experimental data and the model validity. The analysis of variance (ANOVA) of the model demonstrated *p*-value < 0.0001, R^2^ = 0.9923, adjusted R^2^ = 0.9894, and predicted R^2^ = 0.9831. Finally, the predicted film formulation containing 2.75% *w*/*w* hyaluronic acid, 1.321% *w*/*w* Gantrez^TM^ S-97, and 1.246% *w*/*w* pectin was used for further fabrication of MNs (525.4 ± 3.8 µm height and 157.4 ± 2.0 µm base width), which possessed 1508.2 ± 66.2% swelling, with 124.6 ± 7.4 µL of collection volume, and could withstand thumb pressure. Moreover, almost 50% of MNs achieved a skin insertion depth of approx. 400 µm, with 71.8 ± 3.2% to 78.3 ± 2.6% recoveries. The developed MNs show a promising prospect in microsample collection, which would be beneficial for POCT.

## 1. Introduction

The utilization of hypodermic needles for drug administration and sample extraction has a long-standing history. Despite their widespread usage, however, this traditional method has notable drawbacks, including pain, invasiveness, psychological distress, bio-hazardous waste, and the necessity for trained healthcare professionals. In recent times, the development of microneedle (MN) systems has been gaining increasing interest as a more effective approach for sample extraction (blood or interstitial fluid (ISF)) and drug delivery, due to their various advantages over hypodermic needle-based systems. MN systems are characterized by micron-sized needles that offer a minimally invasive and more efficient method of drug delivery and sample extraction [1]. Furthermore, MN-based point-of-care testing (POCT) devices have been receiving considerable attention owing to their high potential for extracting and detecting target analytes present in ISF. Skin ISF is located under the stratum corneum layer and abundant in the dermis layer with a thickness from 1500 to 3000 µm [2], which contains exogenous drugs and clinically relevant biomarkers [3]. Therefore, ISF could be used for drug monitoring or disease diagnosis. Microsampling methods in the invasive forms for ISF collection, including suction blistering, iontophoresis, sonophoresis, and microdialysis, require a long extraction time, expensive equipment, and the assistance of medical professionals. To overcome these limitations, MNs with heights of 50–1500 µm can penetrate through an epidermal layer with a thickness of 50–200 µm to extract ISF from the dermis, with advantages such as being self-applicable with minimal pain and invasiveness.

MNs have been investigated for the delivery of drug molecules or the monitoring of biomarkers [4,5], which can be categorized into solid, hollow, coated, dissolving, porous, and hydrogel-forming MNs [6]. Among them, hydrogel-forming MNs are fabricated by covalently or non-covalently crosslinked three-dimensional networks of hydrophilic polymers. The hydrogel-forming MNs can absorb and swell in water or bodily fluids without dissolving, resulting in the formation of a gel-like substance. This property allows for sustained and controlled drug release. The drug can be loaded into the hydrogel matrix, and it slowly releases after the MNs swell and degrade. The swelling ability of hydrogel-forming MNs has been leveraged for other biomedical applications, such as biosensing. For example, hydrogel-forming MNs have been used as minimally invasive sensors for monitoring glucose levels in diabetic patients [7], alcohol monitoring, carcinoembryonic antigen detection for early breast cancer detection [8], and psoriasis therapy and diagnosis [9]. Moreover, the hydrogel-forming MNs overcome the disadvantages of solid and dissolving MNs, which require a long extraction time, repeated application times, and the assistance of external vacuum or pressure [10,11]. Solid MNs are designed to puncture the skin, creating a micro-incision that can be utilized for the collection of bodily fluids and the delivery of active substances to a targeted area. Dissolvable MNs dissolve upon insertion into tissues, leading to the creation of small openings on the tissue surface, which allow ISF to escape. While this unique property presents significant potential for a wide range of medical applications, particularly in precise drug delivery through the rapid dissolution of polymers, it may limit the applicability of ISF extraction [12,13]. Hollow MNs have the possibility of needle hole blockage. Additionally, porous MNs need a backing layer as a reservoir to store the fluids, and increasing the porosity can decrease the mechanical strength of the MNs. In contrast, coated MNs feature a core that is coated with a surface layer. The coating of the MN typically comprises a blend of drugs and polymers that are utilized for drug delivery purposes. Conversely, the coating resembles a semi-permeable membrane and serves for the collection of bodily fluids for drug/biomarker assay purposes [12]. Moreover, the coated layers on the needles are limited because thickly coated needles limit needle insertion capability into the skin.

The development of hydrogel-forming MNs for health monitoring assay purposes should approach a collection volume of ≥1 µL, with a collection time of ≤20 min; thus, the method should be applied in a simple and rapid manner [14]. Statistical designs in particular response surface methodology (RSM) are effective experimental design methods to assist the optimization of MNs that can be performed with multiple factors and levels. RSM (e.g., Box-Behnken design (BBD), central composite design (CCD)) is commonly employed for the optimization of the formulations, which offers benefits for the study of the interaction between factors and responses [15]. Consequently, the model provides a prediction of optimal experimental conditions. Moreover, the statistical designs substantially reduce the resources, time, and effort needed to develop the formulations [16].

Hydrogels are commonly formulated from a variety of natural polymers, including chitosan, alginate, cellulose, hyaluronic acid, pectin, and starch, as well as synthetic polymers such as polyvinyl alcohol and polyethylene glycol. However, the irreparable impact of synthetic polymers on the environment has driven a recent increase in the use of natural polymers. This is due to their biodegradability, nontoxicity, widespread availability, and cost-effectiveness [17]. Hyaluronic acid and Gantrez^TM^ S-97 have been utilized for hydrogel-forming MNs. Hyaluronic acid is a naturally occurring linear polysaccharide made up of repeating N-acetyl-d-glucosamine and d-glucuronic acid units, which has been widely used for hydrogel formulations in biomedical applications due to its excellent hydrophilicity, biocompatibility, biodegradability, nonimmunogenicity, and nontoxicity [18,19]. Gantrez^TM^ S-97 is a synthetic copolymer of methyl vinyl ether and maleic anhydride (free acid), which is a non-toxic and biocompatible crosslinker with good mechanical strength. It has been widely used in denture adhesives, toothpastes, and mouthwash formulations because of its excellent film-forming ability [20]. Furthermore, pectin could be an additional ingredient for altering the properties of MNs. It is a natural polymer composed of galacturonic acid that has been utilized as a gelling agent in the food industry and biomedical and pharmaceutical fields due to their biocompatibility, biodegradability, and nontoxicity [21].

This study aimed to develop hydrogel-forming MNs for the rapid collection of skin ISF in sufficient volumes by a formulation containing hydrogel with the main polymers of hyaluronic acid and Gantrez^TM^ S-97 with the addition of pectin. A variety of RSM (i.e., BBD, CCD, and optimal discrete model) were employed to study the effects of formulation variables on the significant characteristic of hydrogel film (i.e., swelling ability). Suitable models were evaluated by the analysis of variance (ANOVA). Next, the optimal hydrogel film was prepared according to the predicted values, and hydrogel-forming MNs were further fabricated by a micromolding method. Finally, the MNs were assessed by examining the morphology, mechanical strength, swelling and skin permeation abilities, and recoveries.

## 2. Results and Discussion

### 2.1. Experimental Design

**Figure 1:**
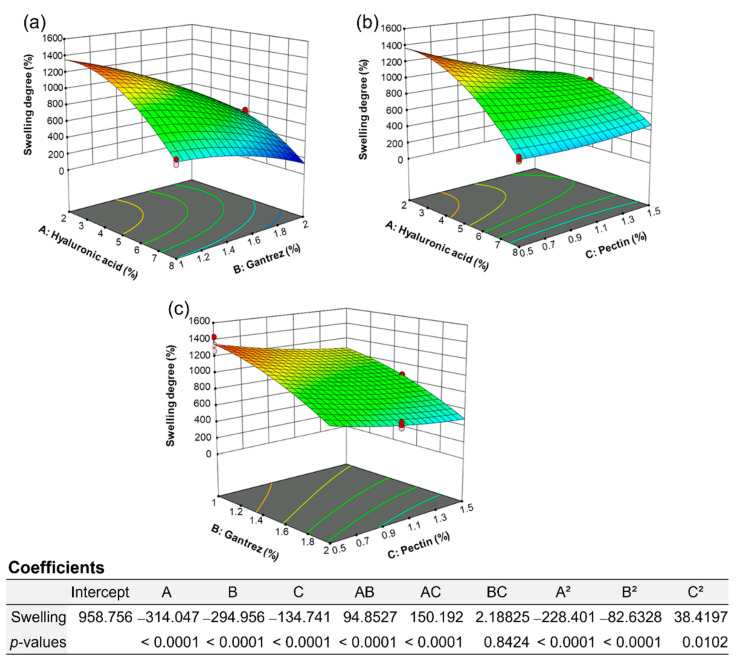
The optimal RSM plots of hydrogel film formulation optimization showing the effect of variables (**a**) hyaluronic acid and Gantrez^TM^ (**b**) hyaluronic acid and pectin and (**c**) Gantrez ^TM^ and pectin on the swelling degree.

The previous report demonstrated the preparation of hydrogel films using 5% *w*/*w* hyaluronic acid and 0.5% *w*/*w* Gantrez^TM^ S-97 as the main polymer and crosslinker, respectively, which yielded a good swelling degree. However, the swelling degree dropped due to incomplete crosslinking of the film caused by an insufficient amount of Gantrez^TM^ S-97. Moreover, Gantrez^TM^ S-97 above 3% *w*/*w* resulted in a decrease in swelling. Moreover, the report suggested the use of 2% and 4% *w*/*w* pectin to enhance the film’s strength, but the results showed a decrease in swelling [22,23]. Therefore, RSM was employed for the optimization of hydrogel film formulations in order to obtain the appropriate swelling degree and strength; RSM generated the mathematical relationship between the independent variables (i.e., 2–8% *w*/*w* hyaluronic acid, 1–2% *w*/*w* Gantrez^TM^ S-97, and 0.5–1.5% *w*/*w* pectin) and the dependent variable (i.e., swelling degree). The experimental data obtained from the hydrogel film formulations listed in Table S1 (in Supplementary Materials) were utilized to create three models, including BBD, CCD, and the optimal discrete models. The appropriate model was chosen by the evaluation of different parameters obtained from regression analysis such as *p*-value, adjusted R^2^, predicted R^2^, and predicted residual sum of square (PRESS) value. Moreover, the ANOVA was applied to examine the significance of the model. All models were significant for the swelling degree with *p*-values < 0.05. The greater adjusted R^2^, predicted R^2^, and low PRESS value indicated the good fit of the model; therefore, the optimal (discrete) model was selected (Table 1). The quadratic equation generated from the models (Figure 1) is as follows: Y (% swelling) = 2400.08714 − 45.88457X_1_ + 76.75263X_2_ − 1090.60741X_3_ + 63.23516X_1_X_2_ + 100.12782X_1_X_3_ + 8.75302X_2_X_3_ − 25.37784(X_1_)^2^ − 330.53100(X_2_)^2^ + 153.67881(X_3_)^2^, where Y = % swelling, X_1_ = hyaluronic acid concentration (% *w*/*w*), X_2_ = Gantrez^TM^ S-97 concentration (% *w*/*w*), and X_3_ = pectin concentration (% *w*/*w*). In addition, the coefficient values in Figure 1 illustrated the synergistic (positive sign) and antagonistic (negative sign) effects of variables. The alteration of hyaluronic acid and pectin concentrations were the most influential factors on the swelling degree. The optimized hydrogel film formulation predicted from the model consisted of 2.75% *w*/*w* hyaluronic acid, 1.321% *w*/*w* Gantrez^TM^ S-97, and 1.246% *w*/*w* pectin, offering the swelling degree of 958.76. The model was a good fit for the range of independent variables used to establish the model. However, the relationship between the independent and dependent variables can change outside of this range.

### 2.2. Hydrogel Films

**Figure 2:**
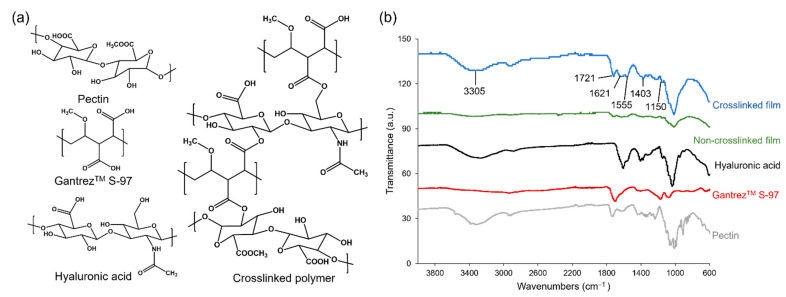
(**a**) The structures and (**b**) IR spectra of hydrogel ingredients, non-crosslinked, and crosslinked hydrogel films.

**Figure 3:**
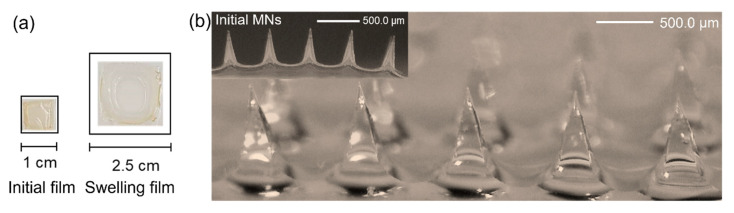
The dry and swelling forms in the artificial ISF for 5 min of (**a**) hydrogel films and (**b**) hydrogel-forming MNs.

The predicted value of each variable was used to formulate hydrogel films, which demonstrated 8.27 ± 0.32 mm length, 8.30 ± 0.17 mm width, and 0.20 ± 0.00 mm thickness. The Fourier transform infrared (FTIR) spectra of hydrogel ingredients and corresponding non-crosslinked and crosslinked films are shown in Figure 2. Hyaluronic acid, Gantrez^TM^ S-97, and pectin were utilized as the main polymer, crosslinking agent, and film-strength enhancer, respectively. The crosslinked film by esterification showed peaks at 1721 and 1150 cm^−1^, which are assigned to the C=O stretch and the C-O stretch, respectively. The peak at 3450 cm^−1^ is associated with free -OH groups of ingredients. The amide groups of hyaluronic acid show the peak of C = O stretch at 1555 and 1621 cm^−1^ and C-N stretch at 1403 cm^−1^. The peaks representing the C=O stretch and the C-O stretch were found to decrease when the film was not crosslinked. The swelling degree of the hydrogel films was 1033.5 ± 25.7% after placing them in the artificial ISF for 5 min (Figure 3), which was slightly greater than the predicted value of 958.76. This film formulation was further utilized for the fabrication of MNs.

### 2.3. Fabrication and Characterization of Hydrogel-Forming MNs

The hydrogel-forming MNs featuring 100 pyramidal-shaped needles were fabricated by a simple micromolding method. The height and base width of MNs were 525.4 ± 3.8 µm and 157.4 ± 2.0 µm, respectively, which would penetrate through the epidermis (50–200 µm thickness) and reach the dermis (1500–3000 µm thickness) for the microsampling of ISF [2]. The reduction in the height of the MNs, as compared to the micromold (600 µm), can be attributed to the viscosity of the hydrogel formulation. This viscosity may limit the flowability and distribution of the hydrogel in the mold cavity. Furthermore, hydrogel is a viscous formulation that generates a high-contact angle, resulting in low wettability [24]. Therefore, the hydrogel may not entirely fill the mold tip, which is the smallest part of the mold. Although a reduction in microneedle height occurred, it remains adequate for effective skin penetration. Furthermore, it is possible to optimize the centrifugation speed and time to overcome the limitations imposed by the viscous formulation.

Then, the MNs were placed in the artificial ISF for 5 min, which demonstrated the swelling degree of 1508.2 ± 66.2% and the collected volume of 124.6 ± 7.4 µL (Table 2). After removal from the ISF, the MNs remained in the pyramidal shape (Figure 3).

**Figure 4:**
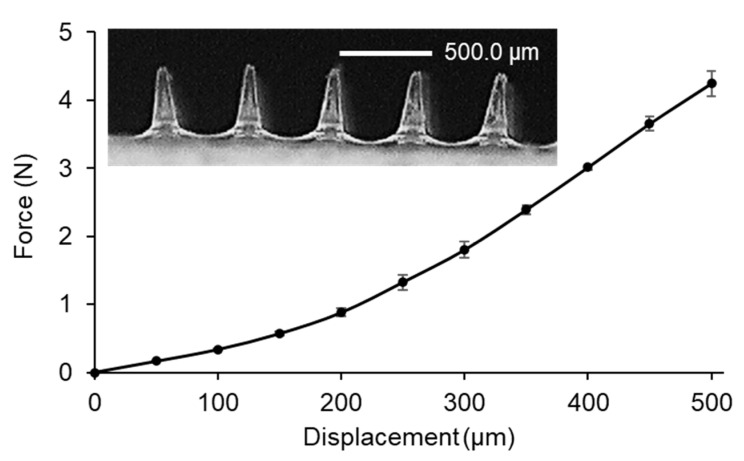
Force-displacement curve from mechanical strength test of hydrogel-forming MNs.

**Figure 5:**
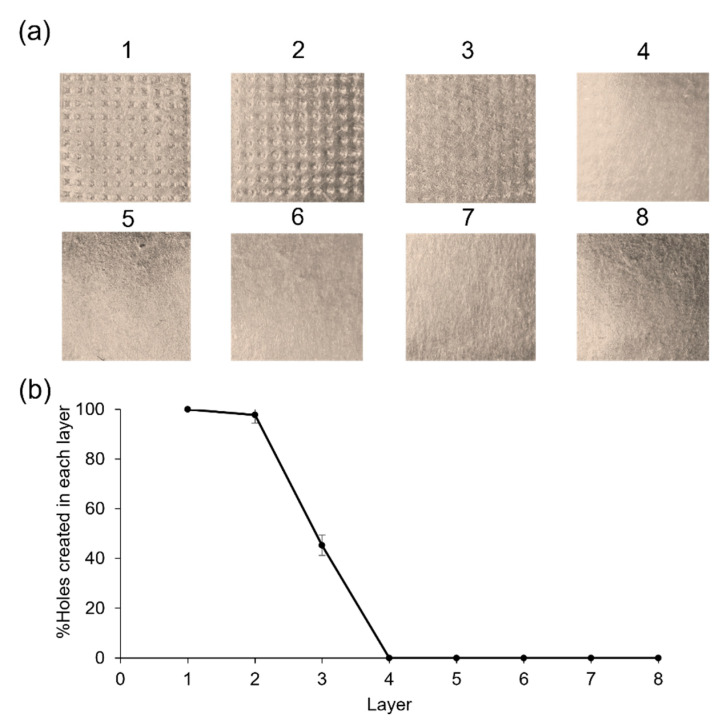
(**a**) Skin insertion test of hydrogel-forming MNs on (**b**) model skin (PF) layers.

In general, MN patches can be adhered to the skin by applying pressure with the thumb. Therefore, the mechanical strength test of MNs was performed in the compression mode using the constant force of 32 N per array for 30 s, mimicking thumb pressure. The bluntness of the MN tips was associated with a steep slope in the force-displacement curve (Figure 4) [25]. However, the MNs were strong enough to withstand the pressure [26,27]. Next, the Parafilm^®^ M (PF) membrane was used for MN testing as a stratum corneum simulant, which is the outermost layer of the epidermis. In order to investigate the influence of the manual application force in the MN insertion, the created holes on an eight-stacked PF layer were examined. The created holes of 100.0 ± 0.0%, 97.7 ± 3.2% and 45.3 ± 4.2% were observed for the first, second, and third layer, respectively (Figure 5). After compression studies, the percentage height reduction was 11.0 ± 0.5%. The results suggested that the MNs were capable of skin insertion into the dermis layer since 100% of MNs achieved an insertion depth of 268 µm, and almost 50% of MNs achieved an insertion depth of 402 µm. Although the PF had significantly lower insertion depths than neonatal porcine skin, the differences were typically less than the 10% of the total needle length [28]. The developed MNs would achieve deeper insertion in the ex vivo test.

To study in vitro recovery of the extracted ISF extraction, the calibration curve of methylene blue in ISF was established in the range of 0.3125–5 µg/mL. The linear equation was y = 3459.5x + 3230 with correlation coefficient (*r*) of 0.9966. The MNs were soaked with 0.3125, 1.25, and 5 µg/mL of the methylene blue. The percent recovery of the extracted ISF was in the range of 71.8 ± 3.2 to 78.3 ± 2.6%, indicating that the MNs were able to absorb the sufficient ISF volume.

The model suggested the appropriate mixture of ingredients for the formulation of hydrogel-forming MNs, which assisted to shorten the experimental time. Compared to the previous reports, hydrogel-forming MNs exhibited enhanced swelling properties attributed to the incorporation of optimal Gantrez^TM^ S-97. This inclusion facilitated the preservation of swelling degree, precluding their decrease during ISF collection caused by MN dissolution [22]. In addition, the model-predicted value for the addition of pectin improved the strength of the hydrogel-forming MNs, while maintaining excellent swelling properties. Also, the MNs were able to withstand thumb pressure and effectively penetrate the skin [23].

## 3. Conclusions

Experimental design using the mathematical model RSM was efficient for the optimization of hydrogel film. The optimal discrete model was chosen for the prediction of the appropriate value of each ingredient in the formulation according to high R^2^ and low PRESS. Consequently, the effects of the hydrogel formulation variables on the film characteristic can be easily predicted and precisely interpreted by using mathematical equations. The suggested hydrogel film formulation for further MN fabrication contained 2.75% *w*/*w* hyaluronic acid, 1.321% *w*/*w* Gantrez^TM^ S-97, and 1.246% *w*/*w* pectin. The MNs displayed excellent mechanical properties for adequate insertion without breaking. All MNs exhibited the potential to penetrate the epidermis layer with an insertion depth of approx. 270 µm. Additionally, approx. 50% of the MNs achieved a skin insertion depth of approx. 400 µm. Also, the MNs showed good swelling with ISF upon insertion and rapid and passive extraction within 5 min. The proposed hydrogel-forming MNs could potentially be employed for minimally invasive microsampling and extraction of dermal ISF, which is a source of biomarkers or exogenous drugs.

## 4. Materials and Methods

### 4.1. Materials and Chemicals

Hyaluronic acid (MW 420 kDa) was purchased from Nanjing Gemsen International Co., Ltd., Nanjing, China. Gantrez^TM^ S-97, a copolymer obtained from the free acid of methyl vinyl ether and maleic anhydride (PMVE/MA) (MW 1500 kDa), was provided by Ashland, Worcestershire, UK. Pectin from red apples was purchased from Chemipan, Bangkok, Thailand. MN molds (600 µm height, 200 µm base width, 500 µm tip to tip interspace, 10 × 10 pyramidal arrays) were purchased from Micropoint Technologies Pte Ltd., Singapore. Other chemicals used were analytical grade.

### 4.2. Experimental Design

A randomized RSM, including CCD, BBD, and optimal (discrete) design, was used to study the effect of three variables on hydrogel films’ swelling properties. The dependent and independent variables, along with their levels, are listed in Table 1. A suitable model was chosen by assessing various parameters from the regression analysis and ANOVA such as *p*-value, adjusted R^2^, predicted R^2^, and PRESS values. The predicted optimal condition was tested experimentally in triplicate to validate the results.

### 4.3. Hydrogel Films

#### 4.3.1. Preparation Procedure

Ten grams of each aqueous formulation (Table S1) (in Supplementary Materials) were prepared by homogenous mixing at 300 rpm. The mixtures were centrifuged (Universal 320, Andreas Hettich GmbH & Co. KG, Tuttlingen, Germany) at 4500 rpm for 15 min to remove air bubbles. Then, each formulation (0.30 ± 0.03 g) was separately cast into polydimethylsiloxane (PDMS) square-shaped molds (10 × 10 mm) and dried at room temperature for 18 h prior to peeling off the dried films. After that, the dried films were crosslinked in the oven at 80 °C for 24 h. Finally, the crosslinked films were stored in the desiccator for further analysis.

#### 4.3.2. Characterization

The morphology, including length, width, and thickness of hydrogel films, was measured by the digital vernier caliper. Then, FTIR analysis of all ingredients and non-crosslinked and crosslinked films were carried out in the range of 4000–600 cm^−1^ (iS5 FTIR spectrometer with iD7 attenuated total reflection), Thermo Fisher Scientific, Waltham, MA, USA).

Next, the swelling test of the crosslinked hydrogel films was performed in artificial ISF (pH 7.4) [29]. An empty weighing boat was placed on the analytical balance (LAB 214i, Adam Equipment, Oxford, CT, USA) before transferring the hydrogel film, and the total weight was recorded. Then, 3 mL artificial ISF was added and the total weight recorded. At 5 min, the ISF was taken out, and the weight of the swollen film and the weighing boat was recorded. The swelling degree (%) of the hydrogel films was calculated by the following equation [30]:

Swelling degree = (m_t_ − m_0_)/m_0_ × 100

where m_0_ is the weight of the initial film, and m_t_ is weight of the swollen film at 5 min.

### 4.4. Fabrication Method of Hydrogel-Forming MNs

All the PDMS molds with 100 MNs obtained from the commercial molds were cleaned with the mixture of ethanol and water (1:1, *v*/*v*) by sonication for 20 min. The optimal formulation (0.04–0.05 g) was filled into the molds and centrifuged at 4500 rpm for 15 min at room temperature. Next, the molds were filled up to the weight of 0.150 ± 0.015 g. The MNs were dried at room temperature for 18 h, crosslinked at 80 °C for 24 h, and kept in the desiccator for further analysis.

### 4.5. Characterization of Hydrogel-Forming MNs

#### 4.5.1. Morphology

The images of MNs were taken by a digital microscope (Dino-Lite Edge AM4115T-YFGW, AnMo Electronics Corporation, Taipei, Taiwan) that was capable of variable magnification of 20–220×. The accurate measurement of height and base width of the MNs was carried out by adjusting the magnification and focal height and setting the calibration.

#### 4.5.2. Swelling Test

The swelling test procedure of the hydrogel-forming MNs was performed in the same manner as that of the hydrogel films. Then, the volume of ISF absorbed into each MN was also determined using the artificial ISF (density of 1.005 ± 0.001 g/mL). Also, the morphology of the MNs was evaluated before and after the swelling test.

#### 4.5.3. Mechanical Strength Test

The mechanical strength of hydrogel-forming MNs was investigated by the TA.XTplusC texture analyzer (Stable Micro Systems, Godalming, UK) in the compression mode. The MNs were attached to the cylindrical probe (P/0.5, 2.45 cm diameter cylinder) by double-sided tape. The probe was lowered at a speed of 0.5 mm/s (pretest speed 1 mm/s and post-test speed 10 mm/s, trigger force 0.049 N) until a pre-set force of 32 N, mimicking thumb pressure, was achieved. Subsequently, the force was analyzed to obtain a force-displacement curve. Furthermore, the height of MNs before (H_a_) and after (H_b_) the application of compressive force was measured, using the digital microscope. The percentage of height reduction of MNs was calculated by the following equation [30,31]:

% Height reduction = (H_a_ − H_b_)/H_a_ × 100%

#### 4.5.4. Skin Insertion Test

The skin insertion test was performed using the validated skin model, PF, and the texture analyzer. A stack of 8 layers total of PF (134 ± 15 µm thickness for each layer) was placed on the base of the test machine, and the hydrogel-forming MNs were attached to the cylindrical probe. The procedure and all parameters were the same as mentioned in Section 4.5.3. The inserted MN arrays were removed after holding for 30 s. Then, the PF layers were unfolded, and the number of holes produced in each layer were counted and evaluated using the digital microscope. The percentage of holes in each layer generated by MNs was calculated by the following equation [28]:

% Holes in PF layer = number of holes/number of MNs in the array × 100

#### 4.5.5. In Vitro ISF Extraction and Recovery Test

A calibration curve was established by plotting the fluorescence intensity against five different concentrations of methylene blue in the artificial ISF (0.3125–5 µg/mL). Then, the hydrogel-forming MNs were weighed and placed in the ISF containing 3 concentration levels (low, medium and high) of methylene blue for 5 min. The MNs were taken out and weighed before transferring to a centrifuge tube containing 3 mL ISF. After centrifugation at 5000 rpm for 5 min, the supernatant was spectrofluorimetrically measured at the excitation wavelength of 650 nm and the emission wavelength of 685 nm. The percentage of recovery was calculated with the following equation:

% Recovery = found concentration/added concentration × 100

### 4.6. Statistical Analysis

The RSM models employed Design-Expert software (Version 13, Stat-Ease Inc., Minneapolis, MN, USA). All experiments were performed in triplicate. The data were expressed as mean ± standard deviation.

## Figures and Tables

**Table 1:** Fit statistics of the models.

Model	Variables and Level	F-Value	*p*-Value	R^2^	Adjusted R^2^	Predicted R^2^	Adeq Precision	PRESS
BBD	2–8% *w*/*w* Hyaluronic acid, 1–2% *w*/*w* Gantrez^TM^ S-97, and 0.5–1.5% *w*/*w* Pectin	120.34	<0.0001	0.9722	0.9641	0.9505	32.71	2.35 × 10^5^
CCD		258.73	<0.0001	0.9894	0.9856	0.9784	49.46	8.81 × 10^4^
Optimal discrete		341.94	<0.0001	0.9923	0.9894	0.9831	59.12	6.83 × 10^4^

**Table 2:** Characteristics of the optimal hydrogel-forming MNs.

Characteristics	Value
Shape	Pyramidal
Dimension (height × base width)	525.4 ± 3.8 × 157.4 ± 2.0 µm
Swelling ability	1508.2 ± 66.2%
Collection volume	124.6 ± 7.4 µL
Recovery	71.8 ± 3.2% to 78.3 ± 2.6%
Skin insertion	Up to approx. 400 µm
Mechanical strength	Withstands pressure from a finger

## Data Availability

The data presented in this study are available on request from the corresponding author.

## Supplementary Materials

The following supporting information can be downloaded at: https://www.mdpi.com/article/10.3390/gels9040306/s1, Table S1: Hydrogel film formulations.
