# Supplementary material for: Response Surface Methodology for Optimization of Hydrogel-Forming Microneedles as Rapid and Efficient Transdermal Microsampling Tools
Source: Gels. 2023 Apr 6;9(4):306. doi: 10.3390/gels9040306 (PMC10137625; doi:10.3390/gels9040306)
Supplement: Supplementary file 1 [file gels-09-00306-s001.zip › gels-2317936-supplementary.pdf]

Supplementary Materials

Table S1. Hydrogel film formulations.

Formulation No.	Amount (%w/w)		
	Hyaluronic acid	Gantrez™ S-97	Pectin
1	0.76	1.50	1.00
2	2.00	1.00	0.50
3	2.00	1.00	1.00
4	2.00	1.00	1.50
5	2.00	1.50	0.50
6	2.00	1.50	1.00
7	2.00	1.50	1.50
8	2.00	2.00	0.50
9	2.00	2.00	1.00
10	2.00	2.00	1.50
11	5.00	0.79	1.00
12	5.00	1.00	0.50
13	5.00	1.00	1.50
14	5.00	1.50	0.29
15	5.00	1.50	1.00
16	5.00	1.50	1.50
17	5.00	1.50	1.71
18	5.00	2.00	0.50
19	5.00	2.00	1.00
20	5.00	2.00	1.50
21	5.00	2.21	1.00
22	8.00	1.00	1.00
23	8.00	1.50	0.50
24	8.00	1.00	1.50
25	8.00	1.50	1.50
26	8.00	2.00	0.50
27	8.00	2.00	1.00
28	8.00	2.00	1.50
29	9.24	1.50	1.00
